# Comparison of Vonoprazan and Amoxicillin Dual Therapy with Standard Triple Therapy with Proton Pump Inhibitor for Helicobacter Pylori eradication: A Randomized Control Trial

**DOI:** 10.12669/pjms.38.4.5436

**Published:** 2022

**Authors:** Bader Faiyaz Zuberi, Faiza Sadaqat Ali, Tazeen Rasheed, Nimrah Bader, Sana Muhammad Hussain, Anoshia Saleem

**Affiliations:** 1 Bader Faiyaz Zuberi FCPS, Meritorious Professor, Department of Medicine/Gastroenterology, Dow Medical College, Dow University of Health Sciences, Karachi, Pakistan; 2 Faiza Sadaqat Ali FCPS, Senior Registrar, Department of Medicine/Gastroenterology, Dow Medical College, Dow University of Health Sciences, Karachi, Pakistan; 3 Tazeen Rasheed FCPS, Associate Professor, Department of Medicine/Gastroenterology, Dow Medical College, Dow University of Health Sciences, Karachi, Pakistan; 4 Nimrah Bader MD, PGY-4 Geriatric Medicine Fellow, University of Miami/Jackson Health System, Miami, FL, USA; 5 Sana Muhammad Hussain PG Trainee, Department of Medicine/Gastroenterology, Dow Medical College, Dow University of Health Sciences, Karachi, Pakistan; 6 Anoshia Saleem FCSP (Gastro), Consultant, Dr Ruth KM Pfau Civil Hospital Karachi, Pakistan

**Keywords:** Helicobacter Pylori, Vonoprazan, Proton Pump Inhibitors, Clarithromycin, Amoxicillin

## Abstract

**Objectives:**

To compare the efficacy of Vonoprazan based dual treatment versus PPI based treatment for the eradication of Helicobacter pylori infection.

**Methods:**

A randomized controlled trial was conducted in Department of Medicine/Gastroenterology Ruth KM Pfau Civil Hospital, DMC during the period of 22 June to 21 September 2021. Sample size was calculated as 96 in each Group. All patients of age 18-75 years with Helicobacter Pylori Infection were inducted and randomly allocated to two groups. Group-A: were given Capsule Amoxicillin 1 gm; Tablet Clarithromycin 500 mg; Capsule Omeprazole 20 mg all medications were given twice daily for two weeks. Group-B were given Capsule Amoxicillin 1 gm; Tablet Vonoprazan 20 mg (Vonozon^©^, m/s Getz Pharma, Pakistan) twice daily for two weeks. Confirmation of Hp eradication was done by stool Hp antigen test four weeks after completion of treatment. Nine and four patients were lost to follow-up in Group-A & B respectively. Analysis was conducted on 87 patients in Group-A and 92 patients in Group-B.

**Results:**

Out of eighty-seven patients in Group-A and ninety-two patients in Group-B, 73 (83.9%) patients in Group-A and 86 (93.5%) patients in Group-B had negative H pylori result respectively after treatment (*p* = .042). Significantly higher frequencies of adverse events were observed in Group-A as compared to Group-B in nausea/vomiting (*p* = .035) and bloating (*p* = .045).

**Conclusion:**

VA-dual provides an acceptable eradication rate with fewer adverse events.

## INTRODUCTION

*Helicobacter pylori (Hp) i*s a bacteria that causes inflammation of gastric mucosa and it is a key cause of peptic ulcer globally.^1^ It colonizes the gastric mucosa and is guilty for a number of gastric related diseases such as chronic gastritis, gastric adenocarcinoma, and gastric mucosa associated lymphoid tissue (MALT) lymphoma.^2^ Prevalence of *Hp* varies from 85-95% in developing countries and from 30-50% in developed countries.^3^ It is advised that all patients with evidence of active infection with *Hp* should be offered eradication therapy. Multiple antibiotic regimens have been evaluated for *Hp therapy*. However, there are only few regimens which have consistently achieved high eradication “Initially triple therapy including a proton pump inhibitor (PPI) and two antibiotics such as amoxicillin with clarithromycin or metronidazole was considered as the best therapy for the Hp eradication”. As a result of decreased efficacy of this treatment, other different therapies have been developed.

Major reason for decreased effectiveness was development of resistance to antibiotics and failure to maintain sustained increase in gastric pH of >5.7 during course of the day.^4^-^6^ To overcome this double dose of Proton Pump Inhibitor (PPI) are used in all Hp eradication regimens to maintain high pH. Since PPI based therapy was found to be superior to non PPI based therapy for Hp eradication therefore the addition of anti-secretory drugs to antibiotic is of utmost importance in the eradication regimens.^1^ Vonoprazan (VPZ) is a novel potassium competitive acid blocker (PCAB). It works by competing for potassium on the luminal side of the parietal cell and causes rapid and reversible inhibition of H-K ATPase and therefore inhibits extended acid secretion. In contrast to PPIs Vonoprazan is a more potent inhibitor of acid secretion. It has a rapid onset of action, less anti-secretory variability, greater safety and better tolerability.^1^

Important factors in failure of Hp therapy is local resistance to antibiotics used and if they are taken within two weeks of start of Hp eradication regimen. Clarithromycin resistance is reported at 43.9%^7^ and is a major cause of treatment failure. That’s why we excluded it from our current study and planned to compare standard PPI based triple therapy with Vonoprazan based dual therapy without Clarithromycin. No local studies have compared the efficacy in the eradication rates of Vonoprazan based dual therapy versus PPI based triple therapy for the eradication of Hp infection in our region. Therefore, this study was conducted to compare the efficacy of Vonoprazan based dual therapy with one antibiotic versus PPI based triple therapy with two antibiotics for the eradication of Hp in a randomized control setting.

Our objective was to compare the efficacy of Vonoprazan based dual treatment versus PPI based treatment for the eradication of Helicobacter pylori infection.

### Operational Definition:

Helicobacter Pylori infection: Hp infection will be labelled by any one of following investigation:

- Helicobacter Pylori Stool Antigen
- Histopathology on Giemsa Stain

## METHODS

This Randomized control trial. was conducted in the Medical OPD and Medical Unit-1 of Dr. Ruth K.M. Pfau, Civil Hospital Karachi affiliated with Dow University of Health Sciences from 22 June 2021 till 21 September 2021

Group sample sizes of 96 in Group-A and 96 in Group-B achieve 95.246% power to detect a difference between the group proportions of 0.3. The proportion in Group-A is assumed to be 0.5 under the null hypothesis and 0.8 under the alternative hypothesis. The proportion in Group-B is 0.5. The test statistic used is the two-sided Z-Test with unpolled variance. The significance level of the test is 0.05”. Calculation was done using PASS 2019 Power Analysis and Sample Size Software (2019, NCSS, LLC. Kaysville, Utah, USA, ncss.com/software/pass). The Sampling Technique: ***was*** Non-Probability Consecutive

### Inclusion Criteria:

All patients between age of 18-75 years with Hp infection as described above.

### Exclusion Criteria:

- History of gastric surgery such as partial gastrectomy.
- History of allergy to drugs used in the study.
- Intake of antibiotics, PPIs, corticosteroids within the last two weeks.
- Pregnant or lactating females.
- Alcohol or drug addiction.
- Severe neurological or psychiatric disorder.

### Data collection procedure:

All patients presenting to the OPD or Medical Unit -I of Dr. Ruth KM Pfau, Civil Hospital Karachi and fulfilling the inclusion criteria were included after taking informed consent.

The participants were randomly allocated to two groups with help of random number table and were treated for two weeks.

### Group-A: were given:

- Capsule Amoxicillin 1 gm twice daily
- Tablet Clarithromycin 500 mg twice a day
- Capsule Omeprazole 20 mg twice a day

### Group-B were given:

- Capsule Amoxicillin 1 gm twice daily
- Tablet Vonoprazan 20 mg twice a day (Vonozon^©^, m/s Getz Pharma, Pakistan).
- Confirmation of Hp eradication was done by stool Hp antigen test 4 weeks after completion of treatment.

### Data Analysis:

Data was stratified according to age & gender. Frequency of gender & mean (±SD) of age was compared between groups using χ^2^ test and Student’s t-test respectively. Frequency of Hp eradication between two groups was compared using Pearson’s χ^2^ test. A *p* value ≤.05 was taken as significant.

## RESULTS

**Fig.1:**
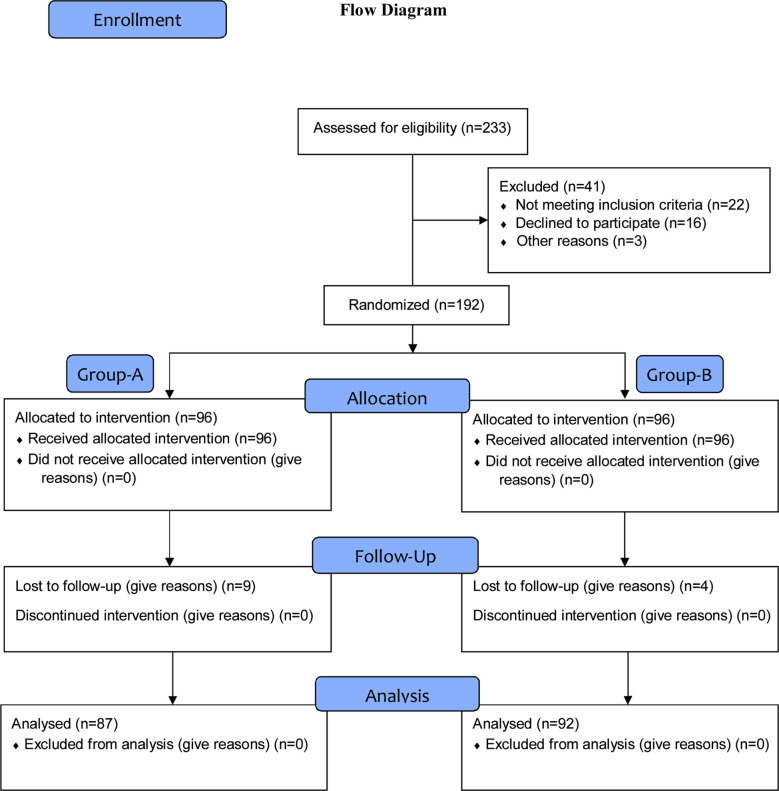
Randomization and Group-Allocation of patients.

Two hundred thirty-three patients were assessed for eligibility out of these a total of 41 patients were excluded. The breakup of these was, not fulfilling selection criteria (n=22), declined to give consent (n=16) and history of using PPI in preceding two weeks (n=3). Randomization was continued till sample size of 96 in each group was achieved. Nine patients were lost to follow-up in Group-A, while four patients were lost to follow-up in Group-B. Analysis was conducted on 87 patients in Group-A and 92 patients in Group-B. Details of enrollment & randomization are given in Fig.1.

Total one hundred seventy-nine patients were analyzed which included 109 males and 70 females. In Group-A there were 54 (62.1%) males and 33 (37.9%) females and in Group-B there were 55 (59.8%) males and 37 (40.2%) females. Both groups were comparable in frequency of gender c^2^ (df=1, n=179) = .09; *p* = .754. Mean age ±SD in Group-A was 40.2 ±9.8 years and in Group-B was 41.4 ±10.6 years respectively, statistically there was no significant difference in age between two groups *t* (177) = -0.81, *p* = .422.

**Table-I:** Helicobacter Pylori Eradication between Groups.

		Group		Statistics		

		Group-A	Group-B	χ^2^	df	p value
HP Stool Ag	Negative	73 (83.9%)	86 (93.5%)	4.126	1	.042
	Positive	14 (16.1%)	06 (6.5%)			

Total		87 (100%)	92 (100%)			

Out of eighty-seven patients in Group-A and ninety-two patients in Group-B, 73 (83.9%) patients in Group-A and 86 (93.5%) patients in Group-B had negative H pylori result respectively after treatment, c^2^ (df=1, n=179) = 4.126; *p* = .042. Vonoprazan based dual therapy more likely to eradicate H. pylori than convention PPI based triple therapy.

Few adverse effects were reported, but none so severe to stop treatment. Significantly higher frequencies of adverse events were observed in Group-A as compared to Group-B in nausea/vomiting and bloating, details are given in Table-II.

**Table-II:** Comparison of Adverse Events between Groups with χ^2^ test.

	Groups		Statistics		

	Group-A	Group-B	χ^2^	df	p value
Diarrhea	9 (10.3%)	3 (3.3%)	3.588	1	.058
Nausea/Vomiting	13 (14.9%)	5 (5.4%)	4.469	1	.035
Bloating	11 (12.6%)	4 (4.3%)	4.008	1	.045

## DISCUSSION

To the best of our knowledge, this is the first randomized control study to show the efficacy and safety of 14 days Vonoprazan amoxicillin dual therapy. Results of our study demonstrated higher H. Pylori eradication rate of 93.5% by Vonoprazan based dual therapy as compared to PPI based conventional triple therapy, which achieved 83.9% eradication rate (p<0.05). In majority of trials a 7-day dual or triple therapy of vonoprazan was given along with either amoxicillin alone or amoxicillin plus clarithromycin to reveal the eradication rate upto 85% to 87%.^8^ No trial extended upto 14 days to document further improvement in efficacy of vonoprazan.

Another important finding in our study was fewer adverse effects and easy tolerability in vonoprazan based dual therapy compared to conventional triple therapy. Only few patients in VPZ group developed nausea/ vomiting, bloating and diarrhea, but none was severe enough to stop regimen. Similarly Qiu-Ju Lyu *et al.*, in their meta-analysis on efficacy and safety of VPZ-based versus proton pump inhibitor-based triple therapy for helicobacter pylori eradication, reported significantly less incidence of adverse effects and well tolerability in VPZ-based triple therapy than that in PPI-based triple therapy (pooled incidence, 32.7% vs 40.5%; OR, 0.71; 95%CI: [0.53–0.95]; *P*<0.05).^9^

Although Vonoprazan does not have anti-microbial properties, this significant superiority of Vonoprazan over PPI for H-Pylori eradication is suggested mainly from more sustained maintenance of gastric PH as VPZ 40 mg daily can maintain pH > 4.0 for > 90% of the day.^10^ The gastric H^+^,K^+^-ATPase is the preferred target for acid suppression. Until recently, the only drug that effectively inhibited this ATPase was the proton pump inhibitor (PPI). PPI is a pro drug, that require acid for its activation. Once acid-activated, PPI bind to ATPase (proton pump) to inhibit acid secretion. But because of short half-life of PPI and continued *de novo* production of the H^+^, K^+^-ATPase, night-time acid secretion is not fully inhibited. Whereas vonoprazan, which is a potassium-competitive acid blocker (PCAB) is an active drug that does not require acid for its activation, has seven hours half-life, bind reversibly with both active H^+^, K^+^-ATPase pumps and Inactive pumps with slow dissociation rate. PPI require meal for its activation while Vonoprazan has no meal dependency. Onset of action of Vonoprazan is within 24 hours, pH >4 sustained over most of the time, including overnight in majority of patients, without tolerance to the drug.^10^,^11^ This rapid, sustained acid control allows antibiotic to work efficiently at optimal PH against H-Pylori organisms.

Antimicrobial stewardship and recognizing optimal treatment and duration of therapy, without unjustified use of antibiotics is imperative in current era of antimicrobial resistance. DY Graham *et al*. in his study brought up the fact that vonoprazan and amoxicillin dual therapy achieved a cure rate of 80%, when given in clarithromycin resistant patients.^12^ He further highlighted that adding clarithromycin to vonoprazan and amoxicillin as a triple therapy in general population approximately added only 12% benefit in cure rate, and 88% of the patients received clarithromycin unnecessarily.^12^ In his study they gave seven days dual treatment with vonoprazan and amoxicillin to achieve a cure rate of 80 %, while in our study we gave dual therapy for extended period of 14 days to achieved 93.5% cure rate.

One of the main strengths of our study is its study design of being randomized control that provides higher level of statistical reliability. A meta-analysis, where 16 studies were selected for quantitative review, had limitation that 15 of 16 studies in their meta-analysis were retrospective and used historical controls with a time-frame shift between the two groups,^13^ whereas our study is prospective study. Almost all the studies done on vonoprazan gave seven days of treatment, in our knowledge, but we gave it for extended 14 days to fully evaluate its efficacy. Although significantly less adverse effects observed in VPZ dual therapy group, but long-term adverse effects need to be explored from more prolong and sustained acid suppression. Future studies, comparing VPZ dual therapy to VPZ triple, concomitant or sequential therapies may be needed to further evaluate improvement in H-Pylori eradication rate and to develop optimal first line eradication regimen with vonoprazan.

## CONCLUSION

In the era of growing antimicrobial resistance, VA-dual therapy is a potential new first- line Hp therapy for cases because it provides an acceptable eradication rate and high safety and will have a potentially less negative impact on future antimicrobial resistance.

### Authors’ Contribution:

**BFZ, FSA, NB:** Substantial contributions to conception and design, or acquisition of data, or analysis and interpretation of data. **TR, SMH, AS:** Drafting the article or revising it critically for important intellectual content **BFZ:** Final approval of the version to be published. **NB:** Statistical Analysis. Agreement to be accountable for all aspects of the work in ensuring that questions related to the accuracy or integrity of any part of the work are appropriately investigated and resolved. (All Authors).
